# Viscoelastic properties of white and gray matter-derived microglia differentiate upon treatment with lipopolysaccharide but not upon treatment with myelin

**DOI:** 10.1186/s12974-021-02134-x

**Published:** 2021-03-29

**Authors:** Thecla A. van Wageningen, Nelda Antonovaite, Erik Paardekam, John J. P. Brevé, Davide Iannuzzi, Anne-Marie van Dam

**Affiliations:** 1 Department of Anatomy & Neurosciences, MS Center Amsterdam, Amsterdam Neuroscience, Amsterdam UMC, Vrije Universiteit Amsterdam, De Boelelaan 1108, 1081 HZ Amsterdam, The Netherlands; 2 Department of Physics and Astronomy and LaserLaB, VU Amsterdam, Amsterdam, The Netherlands

**Keywords:** Biomechanics, Microglia, White matter, Gray matter, Multiple sclerosis, Indentation

## Abstract

**Background:**

The biomechanical properties of the brain have increasingly been shown to relate to brain pathology in neurological diseases, including multiple sclerosis (MS). Inflammation and demyelination in MS induce significant changes in brain stiffness which can be linked to the relative abundance of glial cells in lesions. We hypothesize that the biomechanical, in addition to biochemical, properties of white (WM) and gray matter (GM)-derived microglia may contribute to the differential microglial phenotypes as seen in MS WM and GM lesions.

**Methods:**

Primary glial cultures from WM or GM of rat adult brains were treated with either lipopolysaccharide (LPS), myelin, or myelin+LPS for 24 h or left untreated as a control. After treatment, microglial cells were indented using dynamic indentation to determine the storage and loss moduli reflecting cell elasticity and cell viscosity, respectively, and subsequently fixed for immunocytochemical analysis. In parallel, gene expression of inflammatory-related genes were measured using semi-quantitative RT-PCR. Finally, phagocytosis of myelin was determined as well as F-actin visualized to study the cytoskeletal changes.

**Results:**

WM-derived microglia were significantly more elastic and more viscous than microglia derived from GM. This heterogeneity in microglia biomechanical properties was also apparent when treated with LPS when WM-derived microglia decreased cell elasticity and viscosity, and GM-derived microglia increased elasticity and viscosity. The increase in elasticity and viscosity observed in GM-derived microglia was accompanied by an increase in *Tnfα* mRNA and reorganization of F-actin which was absent in WM-derived microglia. In contrast, when treated with myelin, both WM- and GM-derived microglia phagocytose myelin decrease their elasticity and viscosity.

**Conclusions:**

In demyelinating conditions, when myelin debris is phagocytized, as in MS lesions, it is likely that the observed differences in WM- versus GM-derived microglia biomechanics are mainly due to a difference in response to inflammation, rather than to the event of demyelination itself. Thus, the differential biomechanical properties of WM and GM microglia may add to their differential biochemical properties which depend on inflammation present in WM and GM lesions of MS patients.

**Supplementary Information:**

The online version contains supplementary material available at 10.1186/s12974-021-02134-x.

## Background

Multiple sclerosis (MS) is a chronic, inflammatory neurological disorder, most commonly diagnosed in young adults. Pathologically, it is characterized by demyelination in the central nervous system (CNS) accompanied by inflammation and axonal degeneration [1]. Demyelinated lesions in the white matter (WM) feature microglia with an activated phenotype in addition to infiltrated peripheral leukocytes. In contrast, gray matter (GM)-demyelinated lesions are considerably less inflammatory, with little microglial activation, and are almost devoid of infiltrating leukocytes [2, 3]. Recent studies have shown that microglia in WM and GM in human post-mortem MS tissue show differential gene expression [4], microglia homeostasis, and activation markers [3, 5, 6]. These data suggest that microglia in WM and GM could either represent intrinsic different subpopulations or are distinguished by differences in the local environment, i.e., presence or absence of neurons or inflammation, as visible in MS.

Along with biochemical characteristics, additional aspects relevant to cell physiology and function are biomechanical characteristics of cells and their microenvironment such as the presence of extracellular matrix (ECM) proteins [7, 8]. That the local environment can impact local tissue biomechanical properties is exemplified by measurements of WM and GM brain tissue where the WM is significantly less elastic than the GM [9]. Using magnetic resonance elastography (MRE), a method that can be applied in living patients, a decrease in viscoelastic parameters in WM of MS patients compared to control subjects has been observed [10–12]. Interestingly, even patients presenting with a clinically isolated syndrome (CIS), before a definite diagnosis of MS is reached, showed reduced viscoelasticity of the WM as measured using MRE [10]. In experimental autoimmune encephalomyelitis (EAE), an animal model for MS, an observed decrease in WM elasticity coincides with an increase in local inflammation [13]. In addition, post-mortem analysis of human MS material revealed that active WM lesions featuring a large number of microglia/monocytes presented with a soft mechanical signature as indicated by a decreased elasticity, whereas chronic-active WM lesions, featuring more astrocytes, were considerably stiffer indicated by an increase in elasticity [14]. These results suggest that the local cellular composition and concomitant microenvironment could determine the biomechanical properties of WM tissue in MS. In contrast to studies on WM tissue, no data is available on the effect of inflammation on biomechanical properties of GM tissue. Though, it has been shown that GM tissue damaged by a stab injury is less elastic than the surrounding healthy GM which is accompanied by increased GFAP immunoreactivity as is also present in WM demyelinated lesions [15, 16].

This raises the question of whether changes in tissue biomechanical properties translate to the differences at the cellular level. A relevant study on a number of immune cells showed that they exhibit a variety of elasticity and viscosity depending on the presence of certain cytokines [17]. Of interest is that changes in cell elasticity have been implicated in increased cell migration [18]. Moreover, inflammatory mediators increase phagocytosis in macrophages which are suggested to be mediated by a reduced cell elasticity [19]. In line with this observation, it has been shown that increased cell elasticity reduces the phagocytic capacity of the cell [20]. Thus, there seems a clear association between cell biomechanical properties and cellular functions relevant to MS pathology. In a recent study, we observed that upon treatment with inflammatory lipopolysaccharide (LPS), a bacterial cell wall component, GM-derived astrocytes reduced their cellular elasticity and viscosity, whereas WM-derived astrocytes did not change [21]. This region-specific biomechanical astrocyte response may be relevant for the observed better remyelination of GM areas than WM areas of the MS brain [22].

In the present study, we aim to determine whether microglial cells, as immune cells of the brain involved in MS pathology in WM and GM [5, 23], alter their biomechanical properties upon either treatment with LPS or after myelin phagocytosis, and how this is related to cell morphology and inflammatory status of the microglial cells. To this end, we determined the elasticity and viscosity of microglia derived from WM and GM using indentation. We hypothesize that the biomechanical properties of WM- and GM-derived microglia may contribute to the differential microglial response as seen in MS WM and GM lesions.

## Methods

### Isolation, culture, and treatment of primary adult rat mixed glial cells

Animal studies were approved by the National (CCD) and VU University ethical committees on Animal Experimentation (approval number AVD1120020171784) and carried out in strict accordance with their guidelines. Primary glial cells were isolated from adult Wistar rats. It has been shown that the lack of brain environment in vitro, for microglia derived from post- or pre-natal rodents, can affect microglial identity [24, 25]. This suggests that data generated from in vitro cultures is not reflective of microglial function in the CNS. However, with this in mind, our cultures might still reflect a part of the CNS environment: the indented and studied microglia were cultured in mixed glial cell cultures, also featuring astrocytes which are known to maintain microglial identity in culture [26]. In addition, we cultured these primary glial cells from the brains of adult (> 3 months) rats. Thus, cultured glial cells have been in contact with a mature CNS environment, shaping their identity, for a prolonged time.

After sacrifice, the brains were removed and collected in ice-cold 0.6% glucose in Hanks Balanced Saline Solution (HBSS) and kept on ice. Per glial cell isolation, 2–3 rat brains were used. The brainstem and cerebral cortex were dissected representing WM- and GM-enriched areas, respectively. The brain areas were chopped into small pieces and subsequently trypsinized in 0.25% trypsin (Sigma) with 1.25 μg/ml DNA-se (Sigma) in HBSS for 30 min at 37°C with the tube continuously rotating. Then, a culture medium consisting of equal amounts of Dulbecco’s modified Eagle’s medium (DMEM, Gibco, Life Technologies, Breda) and Ham’s F10 nutrient mix (Gibco, Life Technologies, Breda, The Netherlands) supplemented with 10% fetal bovine serum (Gibco, Life Technologies, Breda), 1% penicillin/streptomycin (Invitrogen), and 1% l-glutamine (Invitrogen) was added to deactivate the trypsin. The tissue was further homogenized by mechanical dissociation using increasingly smaller pipets and finally using a glass small bore Pasteur’s pipette. Subsequently, the homogenate was filtered through a 70-μm mesh (Corning). After filtration, the cell suspension was centrifuged at 1200 rpm for 7 min at 4°C. The supernatant was removed, and the cell pellet was washed 3 times in a culture medium. In order to plate primary glial cells, per brain area, they were suspended in 4.8 ml (when 2 rat brains were used for culture) or 7.2 ml (when 3 rat brains were used for culture) culture medium. Of this cell suspension, 450 μl was plated per poly-l-lysin-coated Ibidi μ-dish (35mm) featuring a grid with a 500-μm repeat distance labeled from A-U; 1-20 (Ibidi, Germany). In order to settle the cells, plated cells were left in the incubator for 1 h before 1 ml of fresh culture medium was added to each dish. The culture medium was changed the day after and thereafter every 3 days. Cells were kept in culture for 7 days before being treated with 100 ng/ml LPS (*E. coli* O55:B5, Difco) in phenol-free culture DMEM (Gibco, Life Technologies, Breda) supplemented with 10% FCS, 1% l-glutamine, and 1% penicillin/streptomycin for either 24 or 48 h or treated with 12.5 μg/ml pHrodo-labeled human myelin with or without 100ng/ml LPS for 24 h or left untreated as a control.

In addition, 450 μl of cell suspension/well were plated on poly-l-lysine-coated ACLAR coverslips (hand-punched from 8 × 10 cm sheets, Electron Microscopy Sciences) in 24-well plates and left in the incubator for 10 min for cells to attach to the coverslip. Then, the medium was gently renewed, and subsequent medium changes and treatment with LPS and/or myelin were similar as described above. Microglia phenotypes were determined on phase-contrast images made during indentation to prevent morphology bias induced by subsequent handling of the cells. The microglial identity of those cells was later confirmed using sequential double-labeling immunocytochemistry for IBA-1 and GFAP as described below.

### Labeling of myelin with pHrodo

Human myelin was isolated and characterized as previously described [27]. Myelin (1 mg/100 μl sterile water, pH=8.3) was labeled using 1 mg/ml pHrodo (a kind gift from Prof. Dr. I. Huitinga, Netherlands Institute for Neuroscience, Amsterdam). Myelin together with pHrodo were incubated at RT on a horizontal shaker for 45 min after which it was centrifuged for 4 min at 12,000*g* at 4 °C. Subsequently, the pellet was washed in Dulbecco’s phosphate-buffered saline (pH = 7.4, dPBS, Gibco) 3 times, each time followed by centrifugation for 4 min at 12,000*g* at 4 °C to eliminate unbound pHrodo, before being suspended in 0.5 ml dPBS at ~2mg/ml.

### Indentation protocol

A detailed description of the indentation protocol and subsequent data analysis have been reported before [21] and is performed identically in the present study. In short, a custom-built indentation arm equipped with a cantilever-based ferrule-top force sensor (Optics11 Life) is mounted on an inverted microscope (Zeiss Axiovert 25, Carl Zeiss Inc.) and enclosed within an acoustically and thermally isolated box with a temperature maintained at 37 °C. Ibidi μ-dishes with cells were placed in the microscope stage and perfused with carbonated phenol-free culture medium (as described above). Each cell was indented once, and the image was saved together with the grid location of the indented cell. All measurements were performed within 3 h after the cells left the incubator.

Force sensors with cantilever stiffness between 0.02 and 0.34 N/m and spherical tip radius between 8.5 and 10.5 μm were used in the experiments. Dynamic indentation measurements consisted of a loading step at 1-μm/s indentation speed followed by a 5-s load relaxation period at fixed indentation depth and subsequent frequency sweep between 1 and 10 Hz at 0.1-μm oscillation amplitude. Storage modulus (elasticity) and loss modulus (viscosity), *E*′ and *E*″, respectively, and damping factor tan(*δ*; viscosity/elasticity) were calculated for each oscillation frequency according to previous sources [28]. To measure the thickness of live cells, we tracked the piezo-transducer elongation upon contact with the substrate and the cell, where the height of the cell is given by the differences between the two distances. The maximum indentation depth varied between 1.6 and 2.6 μm on cells that ranged between 7.6 and 11.3 μm thickness (Suppl. Fig 1). As it is known that indentation depth can influence the storage and loss moduli, storage and loss modulus values were corrected for finite-thickness thin-layer bonded sample effects [29] to eliminate the effects of sensing the stiff substrate underneath the cell (correction factor was ~2).

### Terminology applied to study microglia indentation

From the indentation data on the microglia, the storage and the loss moduli were calculated. Although there are many methods to define elasticity, viscosity, and viscoelasticity [30], in this manuscript, we refer to the storage modulus as the elasticity of cell, whereas the loss modulus will be referred to as the viscosity of cells. Cell elasticity indicates resistance to cell deformation upon a force. When the storage modulus of a cell increases, cell elasticity is increased. Cell viscosity indicates the fluid-like behavior of the cell upon a force. When the loss modulus increases, cell viscosity is increased indicating less fluid-like behavior [7, 30]. Lastly, we calculated the damping factor of cells by dividing the loss modulus by the storage modulus. Cells are defined by their viscoelasticity indicating they are both elastic and viscous. When the damping factor is >1, the cell is more viscous than elastic (viscoelastic liquid), whereas if the damping factor is <1, the cell is relatively more elastic than viscous. This measure can be useful in determining which viscoelastic parameter is more affected by, e.g., a manipulation of the cells.

### Sequential double-labeling immunocytochemistry

To identify the cell type of the indented cells cultured on the Ibidi μ-dishes, primary glial cells were fixed right after indentation with paraformaldehyde (PFA) by adding 8% PFA to an equal volume of phenol-free culture medium to a final concentration of 4% PFA for 20 min. Afterwards, cells were washed 3 times in 1 mM Tris-buffered saline (TBS) and stored in TBS at 4°C until further use. Cells were stained using sequential double-labeling immunocytochemistry as follows: fixed cells were incubated in 1% H_2_O_2_ in TBS for 15 min to block endogenous peroxidase activity. Subsequently, cells were washed in TBS and incubated for 30 min in block buffer consisting of 5% donkey serum in TBS + 0.5% Triton-X. After this blocking step, cells were incubated with the IBA-1 antibody (Table 1) in 5% donkey serum in TBS overnight at 4°C. After incubation with the IBA-1 antibody, cells were washed in TBS and incubated with ImmPRESS anti-Goat IgG Alkaline Phosphatase polymer detection kit (Vectorlabs) for 30 min at room temperature (RT). Subsequently, cells were washed and stained with Liquid Permanent Red (DAKO) to visualize IBA-1 immunoreactivity. After IBA-1 immunoreactivity was apparent, cells were washed in TBS and incubated with the GFAP antibody (Table 1) in 5% donkey serum in TBS for 1 h at RT. After incubation, cells were washed in TBS and incubated in the Envision+ Peroxidase kit for rabbit (Agilent) for 30 min at RT. Subsequently, cells were washed in TBS, and GFAP immunoreactivity was visualized using 3,3-diaminobenzidine (DAB, Sigma, St. Louis, USA) supplemented with imidazole. Afterwards, cells were counterstained using hematoxylin for 1 min RT and were stored in TBS at 4°C.

**Table 1:** Antibodies used for immunocytochemistry

Primary antibody	Dilution	Source (catalog number)	Secondary antibody	Dilution	Source
Enzymatic immunocytochemistry					
Goat x IIBA-1	1:500	Abcam (ab5076)	Impress anti-goat IgG (AP)	Undiluted	Vectorlabs
Rabbit x GFAP	1:2000	DAKO (Z0334)	Envision+ peroxidase kit for rabbit	Undiluted	Agilent
Fluorescent immunocytochemistry					
Goat x IBA-1	1:500	Abcam (ab5076)	AF 488-labeled donkey x goat IgG	1:200	Molecular probes (A11055)
Rhodamine phalloidine (F-actin)	1:80	Life Technologies (R415)	NA	NA	NA

F-actin-binding rhodamine phalloidine is fluorescently labeled

*NA* not applicable

### Fluorescent immunocytochemistry

Fluorescent immunocytochemistry was performed on primary glial cells grown on PLL-coated ACLAR plastic coverslips. After fixation as described above, the fixed cells were incubated in a block buffer consisting of 10% donkey serum in TBS with 0.1% Triton-X for 45 min. After incubation in block buffer, cells were incubated overnight at 4°C with the primary antibodies stated in Table 1 diluted in TBS containing 2% donkey serum with 0.02% Triton-X. Subsequently, cells were washed in TBS and incubated with the corresponding secondary antibodies as stated in Table 1 in diluted block buffer (2% Donkey serum with 0.02% Triton-X) for 1 h at RT. To visualize F-actin, we used directly labeled rhodamine phalloidine diluted in block buffer (Table 1). Cells were incubated with rhodamine phallodine for 1 h at RT. After incubation, the cells were washed in TBS, counterstained with 4′,6-diamidino-2-phenylindole (DAPI), mounted on microscope slides, and embedded with Mowiol. The slides were stored at 4 °C before being imaged using a Leica DM5000 microscope (Leica, Germany) or using a Nikon A1R confocal microscope (Nikon, The Netherlands).

### Semi-quantitative RT-PCR

Mixed glial cells were cultured in a 24-well plate and left untreated (control) or treated with LPS and/or myelin as described above. Per condition, 1–2 wells were lysed in a total volume of 1 ml TRIzol (Invitrogen). To the samples, 200 μl chloroform was added, and tubes were centrifuged at 12,000×*g* for 15 min at 4°C. After the phenol-chloroform extraction, RNA was purified and cleaned up using the E.Z.N.A. MicroElute RNA Clean Up kit (Omega Bio-Tek, Norcross, USA) and analyzed for quality and quantity using a NanoDrop spectrophotometer (Thermo Scientific). The input of RNA for cDNA synthesis for all samples was normalized based on the sample with the lowest concentration of RNA. Per sample, 250 ng total RNA of sufficient quality (260/230 ratio of ≥ 2 and 260/280 ratio ≥1) was reverse-transcribed into cDNA using the High-Capacity cDNA Reverse Transcription Kit (Applied Biosystems, Bleisswijk, The Netherlands) with oligo-d(T) primers (50 μM, Invitrogen) according to the manufacturer’s description. Semi-quantitative RT-PCR was performed in a total volume of 10 μl per sample consisting of 3 μl of Power SYBR Green Master Mix (Life Technologies, Carlsbad, USA), with 50 μM of each forward and reverse primers (Table 2), and 6 ng/μl cDNA in a MicroAmp Optical 96-well Reaction Plate (Applied Biosystems, Foster City, USA). The PCR reaction was performed using the StepOnePlus Real-Time PCR system (Applied Biosystems). The PCR protocol was adapted from the manufacturer’s description and featured 40 cycles with an annealing temperature of 60°C, followed by a melt curve analysis. The relative expression level of the target genes was determined by the LinReg PCR software (version 2014 4.3 (July 2014); website: http://www.hfrc.nl) using the following calculation N0=Nq/*E*Cq (N0=target quantity, Nq=fluorescence threshold value, *E*=mean PCR efficiency per amplicon, Cq=threshold cycle). GAPDH and HPRT1 were selected out of 5 candidate housekeeping genes by NormFinder [31]. Gene expression data (N0 values) were normalized against the mean of *Gapdh* and *Hprt1* N0 values.

**Table 2:** Primers used for qPCR

Gene	Forward primer (5′-3′)	Reverse primer (5′-3′)
*Aif1*	GCCTCATCGTCATCTCCCCA	AGGAAGTGCTTGTTGATCCCA
*Gfap*	CAGACTTTCTCCAACCTCCAG	CTCCTGCTTCGAGTCCTTAATG
*Il-1β*	GCCACCTTTTGACAGTGATG	CTTCTCCACAGCCACAATGA
*Il-6*	CCCCAACTTCCAATGCTCTC	AGATGAGTTGGATGGTCTTGGTC
*Tnfα*	CCACACCGTCAGCCGATT	TCCTTAGGGCAAGGGCTCTT
*Hla-dr*	CACACTTGGAGACCTGGTGAT	AACTCCGCCTGGATGATGGT
*Cd74*	ATGGCTACTCCCTTGCTGATG	GTAGTTCACGGGTCCAGACT
*B2m*	GAGCCCAAAACCGTCACCT	ACCGGATCTGGAGTTAAACTGG
*Gapdh*	GAACATCATCCCTGCATCCA	GCCAGTGAGCTTCCCGTTCA
*Hprt1*	CTCATGGACTGATTATGGACAGGAC	GCAGGTCAGCAAAGAACTTATAGCC

### Statistical analysis

All data were analyzed using SPSS Statistics 22 (IBM, Armonk, USA) or GraphPad Prism 8.2.1 (San Diego, USA). Single-cell indentation data obtained within one culture were considered a repeated measure. We combined data from multiple independent cultures (*N*=2–6). To analyze the data, we used a linear mixed model with culture batch and treatment condition as fixed effects. Post hoc comparisons were made using the Bonferroni correction for experimental conditions (LPS, myelin, and myelin+LPS) compared to control. In addition, since the data were not normally distributed, data were Log10-transformed before statistical analysis. *p* values <0.05 were considered statistically significant.

Semi-quantitative PCR measurements did not show a normal distribution and were, therefore, 10Log-transformed before being analyzed using a repeated measures ANOVA or using a linear mixed model (for *Cd74* mRNA levels). Post hoc testing was performed using Dunnett’s correction to compare the experimental groups to control. *p* values < 0.05 were considered statistically significant.

## Results

### Immunocytochemical characterization of indented cells

After indentation, cells were identified as microglia by immunocytochemical staining for IBA-1 or as astrocytes by staining for GFAP (Suppl. Fig. 2). Cells immunopositive (+) for IBA-1 were considered microglia and were included in the analysis. GFAP^+^ astrocytes and cells that showed no specific staining were removed from all subsequent analyses. Indentation data were obtained from six glial cultures for the untreated and LPS treatment conditions and from two glial cultures for the myelin and myelin + LPS treatment conditions. In total, we analyzed the indentation data obtained from single microglia derived from enriched WM (*N*=130 untreated, *N*=114 LPS-treated, *N*=55 myelin-treated, and *N*=72 myelin+LPS-treated) and enriched GM (*N*=73 untreated, *N*=104 LPS-treated, *N*=37 myelin-treated, and *N*=43 myelin+LPS-treated).

### GM derived microglia are intrinsically less elastic and less viscous than microglia derived from WM

**Fig. 1:**
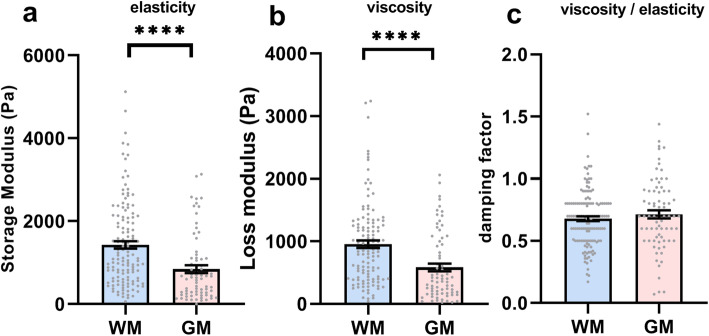
**a** Elasticity (storage modulus), **b** viscosity (loss modulus), and **c** the ratio of viscosity divided by elasticity (damping factor) of microglia derived from WM (blue) and GM (red). Grey dots represent individual microglia measurements from *N*=6 independent cultures. Bars represent the mean of all measurements ± SEM. ****p<0.0001

At baseline, the average elasticity of GM-derived microglia was lower as indicated by a lower storage modulus (mean 842 ± standard deviation 801 Pa) than that of WM-derived microglia (1429 ± 1035 Pa, *t*(201)=4, *p* <0.0001, Fig. 1a). In addition, the viscosity of GM-derived microglia (584 ± 534) was also lower, as indicated by a lower loss modulus, than that of WM-derived microglia (956 ± 658; *t*(190)=4, *p*<0.0001 Fig. 1b). Therefore, GM-derived microglia are less elastic and less viscous than WM-derived microglia. The damping factor of both WM- (0.68 ± 0.22) and GM-derived (0.71 ± 0.28) microglia was similar (*t*(208)=0.967, *p*=0.33, Fig. 1c). Thus, GM-derived microglia are overall less viscoelastic than WM-derived microglia indicating that they are less elastic and more fluid-like.

### WM- and GM-derived microglia show different mechanical properties upon treatment with LPS but not upon treatment with myelin

**Fig. 2:**
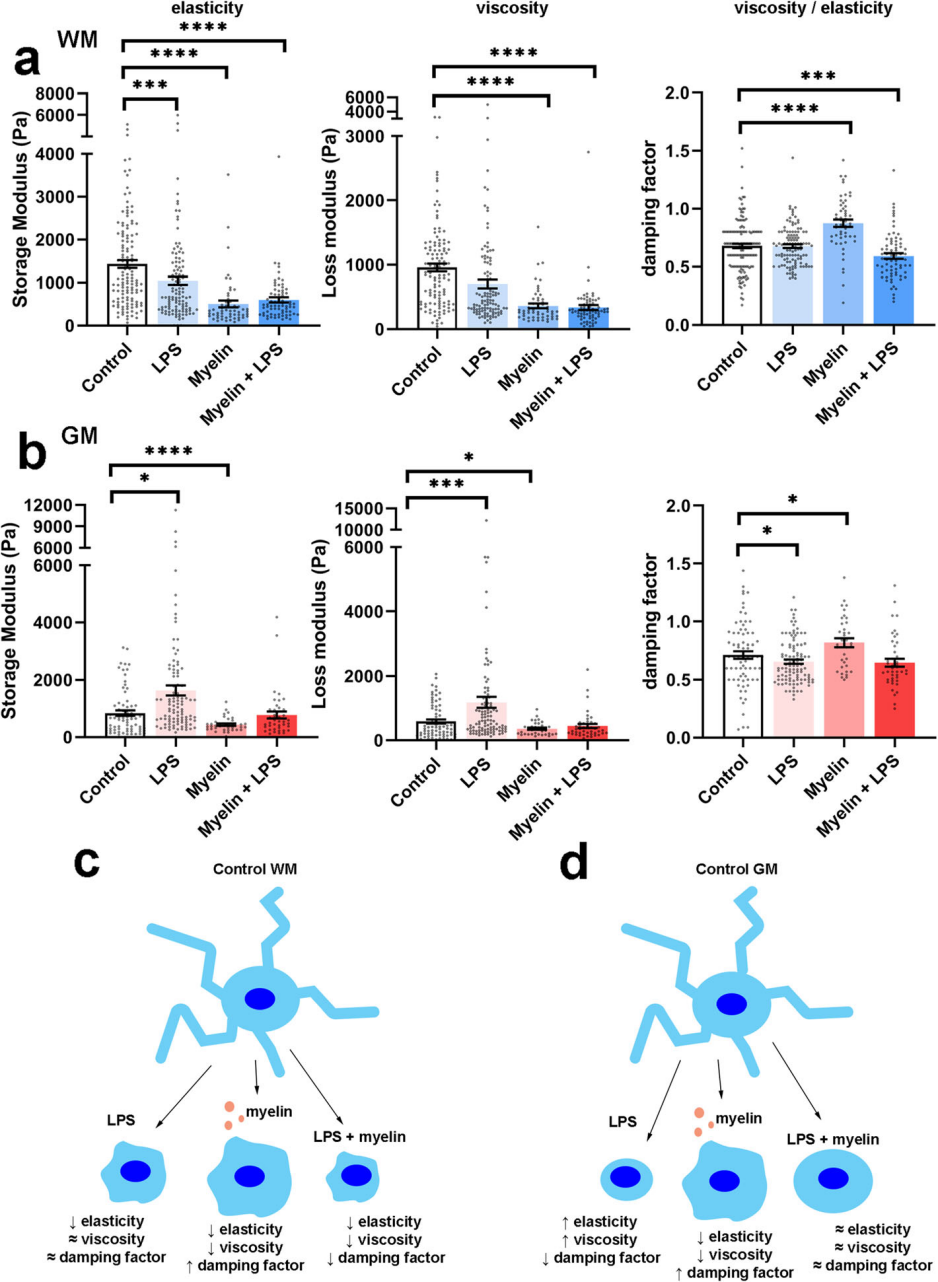
Elasticity (storage modulus), viscosity (loss modulus), and the ratio of viscosity divided by elasticity (damping factor) of microglia derived from **a** WM and **b** GM. Grey dots represent individual microglia measurements from *N*=6 independent cultures. Bars represent the mean of all measurements ± SEM. Schematic overview of the biomechanical changes in **c** WM- and **d** GM-derived microglia after treatment with LPS, myelin, or LPS+myelin. ****p<0.0001

Microglia derived from WM- and GM-enriched brain areas were treated with LPS, myelin, or myelin+LPS for 24 h. Indentation values of single microglia were pooled across separate experiments. WM-derived microglia showed significant changes in elasticity in all treatment conditions in comparison with the control microglia (*F*(3/355)=27.338, *p* <0.0001, Fig. 2a). Treatment of WM-derived microglia with LPS (*p*<0.001), myelin (*p*<0.0001), or myelin+LPS (*p*<0.0001) resulted in a decreased cell elasticity, i.e., lower storage modulus, compared to control microglia which was most pronounced for myelin-treated microglia. GM-derived microglia also showed significant changes in elasticity (*F*(3/243) = 13.560, *p*<0.0001, Fig. 2b) which after post hoc testing shown to be due to significant changes in LPS (*p*<0.05) and myelin-treated (*p*<0.001) microglia in comparison with control microglia. In contrast to WM-derived microglia, GM-derived microglia significantly increased their elasticity, indicated by an increased storage modulus, after treatment with LPS (*p*<0.05). Yet, similar to WM-derived microglia, GM-derived microglia decreased their elasticity after treatment with myelin (*p*<0.0001). Treatment of GM-derived microglia with myelin+LPS did not significantly alter elasticity compared to control microglia (*p*=0.20), probably due to contrasting effects induced by LPS versus myelin exposure. When analyzing cell viscosity, we observed that WM-derived microglia showed significant changes in viscosity (*F*(339)=59, *p*<0.0001, Fig. 2a), which after post hoc testing appeared decreased viscosity, as indicated by a lower loss modulus, compared to control microglia, after myelin (*p*<0.0001) and myelin+LPS (*p*<0.001) treatment. GM-derived microglia also exhibited changes in viscosity (*F*(240)=15.4, *p*<0.001, Fig. 2b) which was significant for LPS-treated cells showing a higher loss modulus indicating increased viscosity (*p*<0.001) compared to control microglia, and myelin treatment resulted in a reduced viscosity (*p*<0.05) compared to control microglia. Treatment with myelin+LPS did not significantly change the viscosity (*p*=0.073).

When determining the damping factor (i.e., viscosity/elasticity) of microglia, we observed that WM- and GM-derived microglia exhibited an altered damping factor (WM: *F*(3/208)=265.7, *p*<0.0001; GM: *F*(3/141)=72.65, *p*<0.0001) upon different treatments. Post hoc testing revealed that upon treatment with LPS, GM-derived microglia showed a decreased damping factor (*p* <0.05, Fig. 2b), whereas WM-derived microglia did not (*p* = 0.99, Fig. 2a). These data indicate that GM-derived microglia treated with LPS showed a relatively larger increase in elasticity than viscosity compared to control microglia. The lack of change in the damping factor of WM-derived microglia treated with LPS indicates that elasticity and viscosity microglia characteristics decreased similarly. Upon treatment with myelin, both WM- (*p*<0.0001) and GM-derived (*p*<0.05) microglia showed a clear increase in their damping factor (Fig. 2 a, b) indicating that the observed decrease in elasticity is larger than the decrease in viscosity (2.0 and 1.6 times, and 2.8 and 2.6 times for WM and GM, respectively) and that cells became relatively more viscous than elastic. In contrast, when treated with myelin+LPS, WM-derived microglia showed a significantly decreased damping factor (*p*<0.001) while elasticity also decreased meaning that the viscous component decreased more than the elastic one (2.8 and 2.4 times, respectively). GM-derived microglia treated with myelin+LPS also showed a slight decrease in the damping factor, but this was not significantly different compared to control microglia (*p*=0.13, Fig. 2b).

From these data, we can conclude that when treated with LPS, WM- and GM-derived microglia show opposing changes in elasticity and viscosity with WM-derived microglia becoming less elastic and less viscous meaning that overall they become more fluid-like. In contrast, GM-derived microglia become more elastic and more viscous with a stronger change in viscosity than elasticity, thus less fluid-like (Fig. 2 c, d). When treated with myelin, both WM- and GM-derived microglia show similar changes, reducing both their viscosity and elasticity (Fig. 2 c, d).

### Elasticity does not relate to microglia morphology

**Fig. 3:**
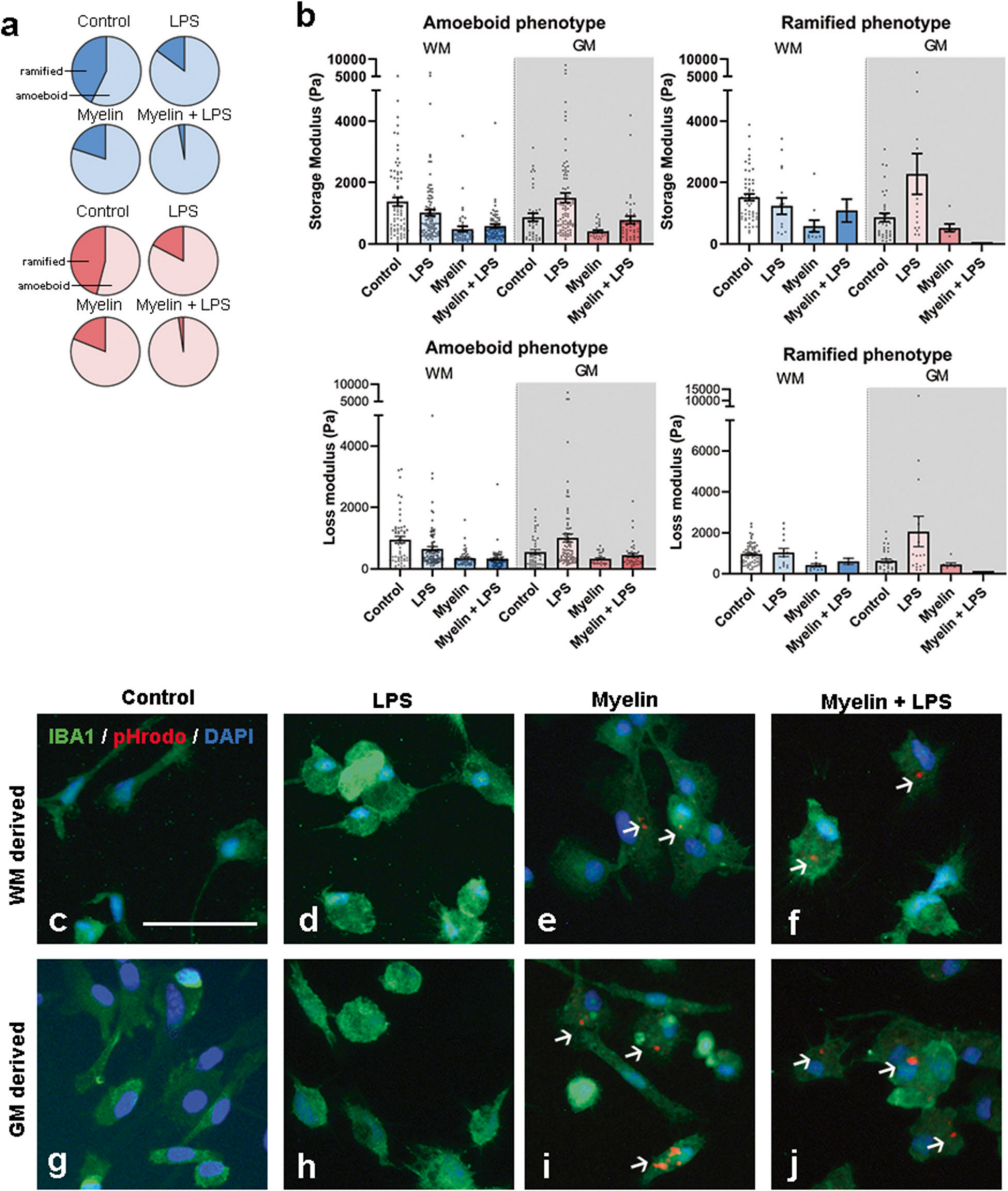
Microglial morphological phenotype does not determine cell elasticity. **a** Pie graphs indicating per condition the percentage of amoeboid and ramified indented microglia per brain area (WM derived = blue, GM derived = red). **b** Elasticity (storage modulus, upper graphs) and viscosity (loss modulus, lower graphs) of the microglia with an amoeboid or ramified phenotype derived from WM (blue) and GM (red). Black dots represent individual microglia measurements from *N*=6 independent cultures for control and LPS conditions and *N*=2 for myelin and myelin+LPS conditions. Bars represent the mean of all measurements per condition ± SEM. Representative immunocytochemical images of IBA-1 (green) in WM-derived microglia (**c**–**f**) and GM-derived microglia (**g**–**j**). Myelin phagocytosis is visualized by pHrodo (red, **e**, **f**, **i**, **j**). Arrows indicate phagocytosed myelin. Scalebar = 50 μm (**c**–**j**)

Changes in elasticity of microglia could be caused or coincide with an altered cell morphology [32]. Therefore, we characterized the morphological appearance of microglia in response to the various treatments. Microglia were categorized as “ramified” if they showed ≥1 ramification or as “amoeboid” when they did not feature any clear ramification. Using this classification, we found that WM- and GM-derived cultures featured a similar ratio of ramified and amoeboid microglia in all treatment conditions (Fig. 3a). In the control condition, only slightly more amoeboid than ramified microglia were present, whereas after treatment with LPS, myelin, or myelin+LPS, most microglia were classified as amoeboid microglia (Fig. 3a). Since we observed similar morphological appearances of WM- and GM-derived microglia treated with either LPS and/or myelin, we questioned if amoeboid microglia exhibit a different cell elasticity than ramified microglia. We, therefore grouped the indentation data according to morphological phenotype and found that the change in elasticity or viscosity after treatment with LPS and/or myelin was similar in amoeboid and ramified microglia (Fig. 3b), and corresponded to the pooled data (Fig. 2a, b). We further studied microglia morphology by visualizing microglia morphology of IBA-1^+^ microglia using immunocytochemical analysis of fixed primary glial cells derived from WM and GM which were not indented. WM- and GM-derived IBA-1^+^ microglia appeared with a similar morphology after treatment with LPS (Fig. 3d, h), i.e., with less ramifications compared to the control microglia (Fig. 3c, g). Microglia derived from WM and GM have slightly more amoeboid morphology when treated with myelin alone (Fig. 3e, i) whereas incubation with myelin+LPS leads to microglia exhibiting a mostly amoeboid morphology (Fig. 3f, j). Although not quantified, we observed that roughly 30–40% of the IBA-1^+^ microglia contained pHrodo-labeled myelin (data not shown).

### Cell morphology, but not cell elasticity or viscosity, relates to overt changes in F-actin labeling

**Fig. 4:**
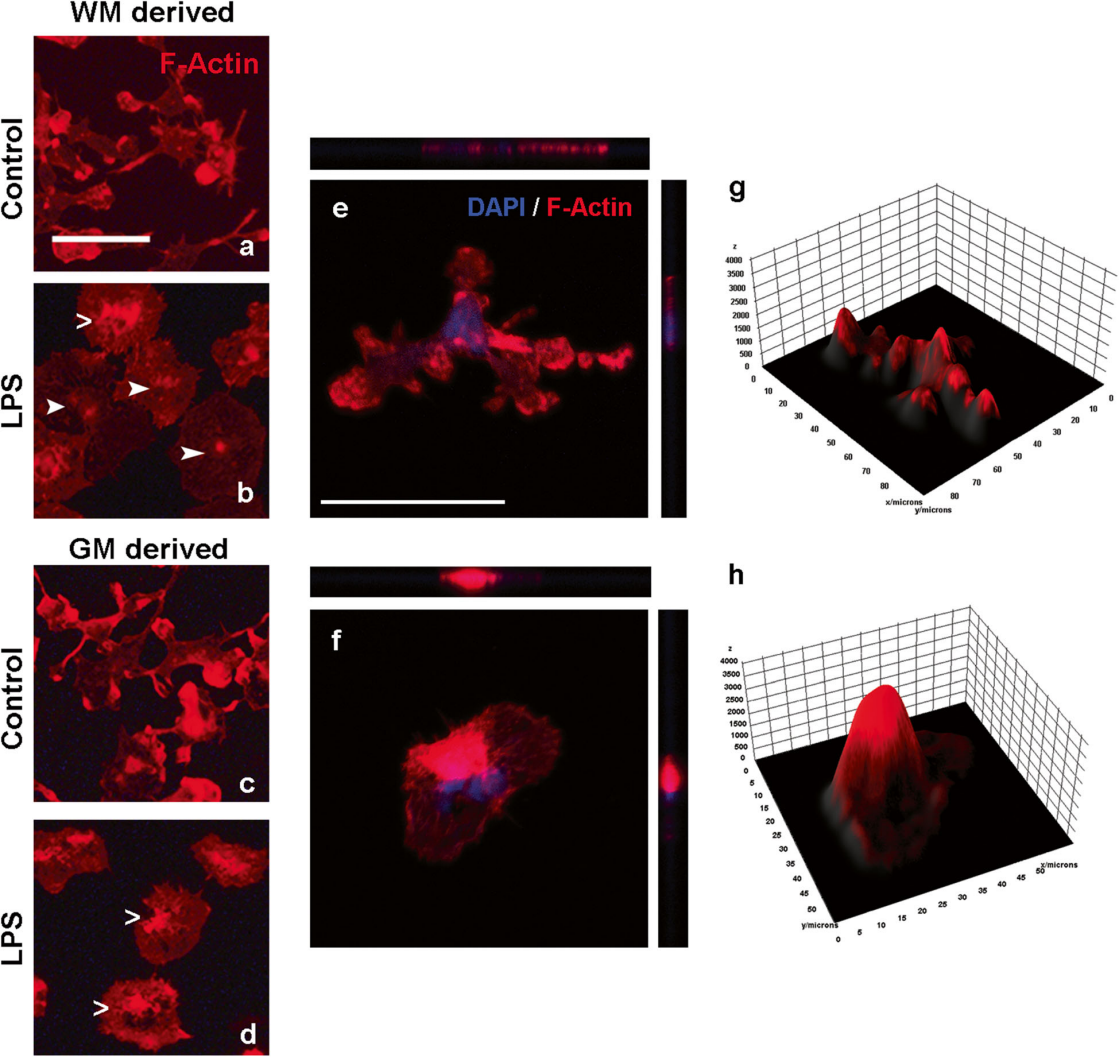
Representative images of F-actin labeling. WM-derived (**a**, **b**) and GM-derived microglia (**c**, **d**) left untreated or treated with LPS. Arrows indicate subtle differences in F-actin signals within the cells treated with LPS: open arrows highlight the stronger F-actin signal localized to the middle of the cell which was visible in nearly all GM-derived microglia treated with LPS and some WM-derived microglia treated with LPS; closed arrows point to a more diffuse and weaker staining of F-actin, visible in WM-derived microglia treated with LPS only. Confocal images with orthogonal slices showing the *z*-axis of a ramified microglia (**e**) and amoeboid microglia (**f**). Surface plots of the same ramified (**g**) and amoeboid (**h**) microglia showing the intensity of the rhodamine phalloidine signal (*y*-axis) which can be considered a measure for the amount of F-actin in the cell and the size of the cells in micrometers (*x*-axis). Scalebars (**a–f**) = 50 μm

In order to elucidate if the differential elasticity of WM- and GM-derived microglia after treatment with LPS was related to a different cytoskeletal rearrangement, we visualized the cytoskeletal protein F-actin in control and LPS-treated WM- and GM-derived microglia only. We found that microglial F-actin expression and organization were similar in WM-derived (Fig. 4a, b) and GM-derived microglia (Fig. 4c, d) reflected by a similar ramified and amoeboid appearance in the control and LPS condition, respectively. However, GM-derived microglia showed a slightly higher abundance of F-actin near the center of the cell than WM-derived microglia after treatment with LPS (Fig. 4b, d). Thus, the differential changes in cell elasticity and viscosity upon treatment with LPS did not associate with overt changes in F-actin expression and organization. Subsequent confocal imaging of F-actin labeling comparing microglia with a ramified and amoeboid phenotype (Fig. 4e, f) showed that F-actin labeling in amoeboid microglia is centered near the nucleus, whereas ramified microglia showed the increased intensity of the F-actin signal near the nucleus and at the end of their processes (Fig. 4g, h).

### GM-derived microglia are more responsive to LPS than WM-derived microglia at the molecular level

**Fig. 5:**
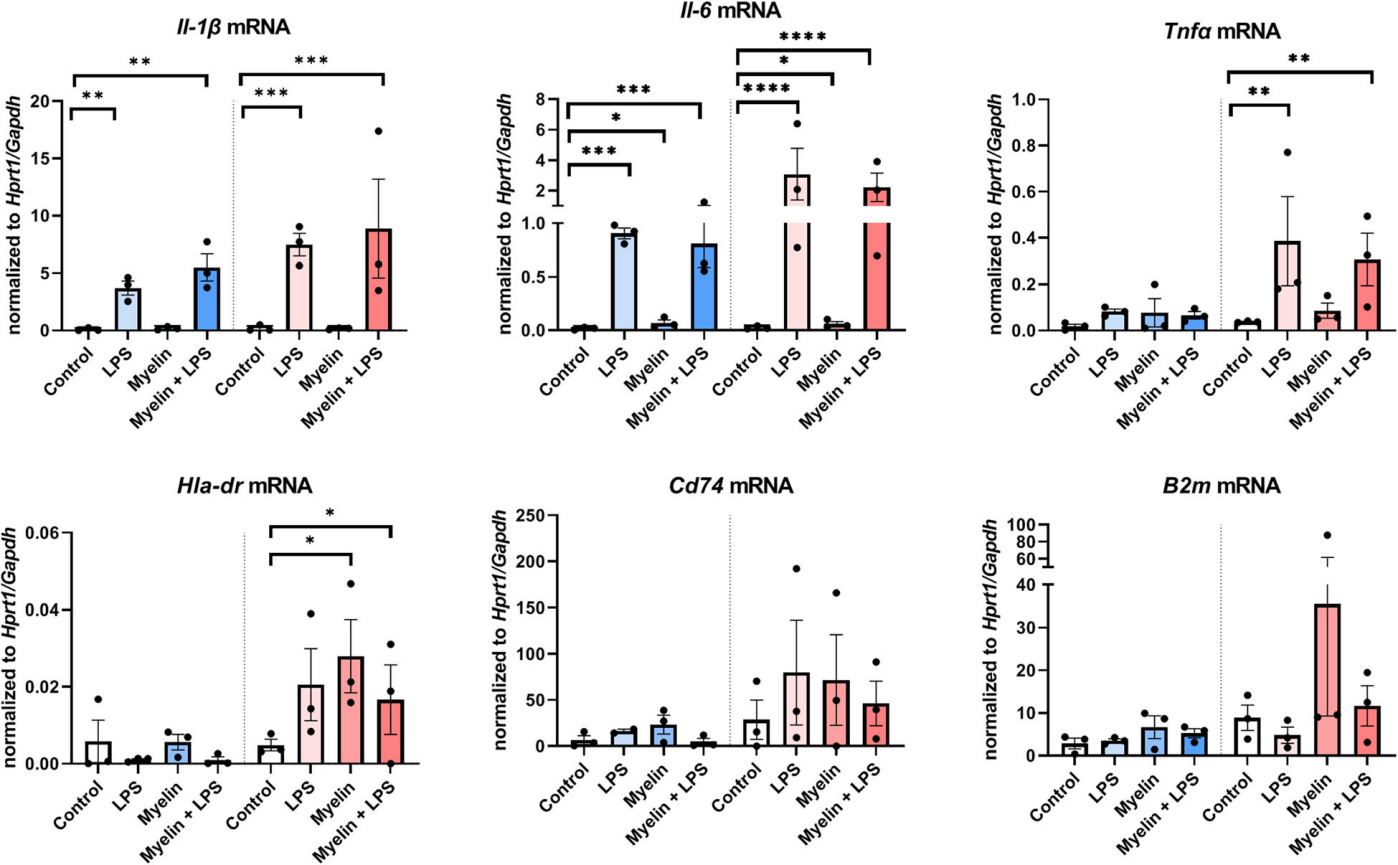
Semi-quantitative mRNA levels of pro-inflammatory cytokines (*Il-1β*, *Tnfα*, and *Il-6*) and of genes involved in antigen presentation (*Hla-dr*, *Cd74*, and *B2m*) in WM-derived microglia (blue, left of dotted line) and GM-derived microglia (red, right of dotted line) upon treatment with LPS, myelin, or LPS+myelin of when left untreated as a control. Data plotted is normalized against the averaged expression of two housekeeping genes (*Gapdh* and *Hprt1*) per sample per condition. Black dots represent data from 3 individual cultures; bars represent the mean ± SEM. *p<0.05, **p<0.01, ***p<0.001, ****p<0.0001

As we found that it was primarily the treatment of microglia with LPS and/or myelin which differentially affected elasticity and viscosity in WM- and GM-derived microglia, rather than indirectly via changes in global morphology, we questioned whether WM- and GM-derived microglia have a differential molecular response towards LPS and/or myelin. As LPS is known to modulate the inflammatory status of microglia, we chose to focus on the gene expression of inflammatory cytokines known to be produced by microglia (*Il-1β*, *Il-6*, *Tnfα* [33, 34]). In order to evaluate the effect of myelin, we chose to focus on the genes of markers involved in antigen presentation expressed by microglia (*Cd74*, *B2m*, and *Hla-dr* [33, 34]), as microglia are considered to be the antigen presenting cells of the CNS and thus could regulate the inflammatory response after phagocytosis of myelin by attracting peripheral lymphocytes into the CNS. By performing semi-quantitative RT-PCR, we first confirmed that our WM- and GM-derived mixed glial cell cultures showed no significant change in *Aif-1* (gene encoding for IBA-1) expressing microglia and *Gfap* expressing astrocytes under the conditions applied (Suppl. Fig. 3). Moreover, *Gfap* expression was relatively absent; thus, we considered the astrocytic contribution to the measured mRNA levels ignorable. *Il1β* expression was enhanced by treatment with LPS or with myelin+LPS in both WM-derived microglia (*F*(3)=16.81, *p*=0.0025) and GM-derived microglia (*F*(3)=31.43, *p*=0.0005) but not by myelin only treatment (Fig. 5). Similar to *Il1β*, *Il6* mRNA was regulated in both WM-derived microglia (*F*(3)=32.60, *p* = 0.0004) and GM-derived microglia (*F*(3)=217.3, *p*<0.0001) showing significant upregulation in all conditions (Fig. 5). In contrast, *Tnfα* mRNA levels were not changed by any of the treatment conditions in WM-derived microglia (*F*(3)=2.589, *p*=0.1483) but were increased in GM-derived microglia (*F*(3)=13.46, *p*=0.0045) treated with LPS or myelin+LPS (Fig. 5). When analyzing antigen presentation-related genes, we observed that WM-derived microglia did not show significant regulation of *Hla-dr* mRNA upon any of the treatments (*F*(3)=0.8473, *p*=0.5164), whereas in GM-derived microglia, *Hla-dr* mRNA levels were increased (*F*(3,5)=6.509, *p*=0.0353) especially in the presence of myelin (Fig. 5). Like *Hla-dr*, *Cd74* and *B2m* mRNA levels were not altered by any treatment in WM-derived microglia (*Cd74*: *F*(3,7)=1.596, *p*=0.2743; *B2m*: *F*(3)= 1.586, *p*=0.2922, Fig. 5). Also, GM-derived microglia did not show significant regulation of *Cd74* (*F*(3,4)=0.6650, *p*=0.6159), but a trend towards increased expression was found in all treatment conditions. *B2m* mRNA did show a significant group difference (*F*(3)=5.976, *p*=0.0311) but no significant difference between conditions compared to control, though a trend towards increased expression was found after exposing microglia to myelin (Fig. 5). These data suggest that the differential response of WM- and GM-derived microglia to the treatment with LPS and/or myelin may be related with changes in cell biomechanics as established for other cell types.

## Discussion

Besides biochemical changes, mechanical properties of glial cells may be involved in the pathogenesis of neurological disorders. In particular inflammatory conditions, as present in MS pathology, can have an impact on the biomechanical changes of cells, as shown for astrocytes [7, 21, 35, 36] and macrophages [19, 32, 37]. The observed difference in microglial activation status in WM lesions versus GM lesions made us question whether differences in biomechanical properties may contribute to this. In the present study, we are the first to show the impact of LPS and myelin treatment on the biomechanical properties of microglial cells. Here, we report that under basal conditions, microglia derived from enriched GM regions show significantly lower elasticity and viscosity than microglia derived from enriched WM regions. As the cells were cultured under the same conditions, this implies that GM- and WM-derived microglia may have different basal intrinsic biomechanical properties. Interestingly, these properties are different than indentation measures of whole GM and WM, where the WM has reduced elasticity and viscosity compared to the GM [9]. This highlights the importance of also investigating individual cell mechanical properties, as these may differ from the mechanical properties of the tissue as a whole, featuring many different cell types and ECM proteins. The elasticity of microglia is comparable to that reported for blood-derived monocytes [32, 37] but is significantly lower than those reported for astrocytes from the same cultures [7, 21, 35, 36]. Furthermore, we report that untreated microglia derived from WM and GM have similar damping factors and similar elastic and viscous properties as other brain cells such as astrocytes and neurons [7]. This relatively soft mechanical signature of microglia, indicated by reduced elasticity and reduced viscosity, compared to astrocytes could be related to the fact that microglia are highly motile cells and able to change morphology drastically and rapidly [38, 39]. As a confounding factor, it has been proposed that the culture substrate could affect the measurement results of cell elasticity and viscosity when indenting to depths which are larger than 5% of the sample thickness [40]. Nevertheless, concurrent with the recently proposed guidelines to eliminate a possible substrate effect [41], we measured microglial cell thickness and corrected our measurements according to the mean cell thickness. In addition, cell measurements where the probe extended into the cell at a depth > 10% of cell thickness were excluded from the analysis.

The observation of a differential biomechanical response of WM- and GM-derived microglia when treated with LPS, but not when treated with myelin, raises the question as to what determines this differential response. Microglia are known to be very plastic cells, changing their morphology from a ramified shape under homeostatic conditions to an amoeboid phenotype during inflammatory conditions. As it has been proposed that the cytoskeleton drives the mechanical properties of cells [35], we studied the F-actin organization in microglia. Interestingly, we found that even though more rearrangement of F-actin cytoskeleton was present in amoeboid compared to ramified microglia, no difference in cell elasticity was observed between the two phenotypes. In addition, WM- and GM-derived microglia presented with similar morphological changes when treated with LPS (i.e., from ramified to amoeboid) but showed opposite effects of LPS on cell elasticity. These results are in contrast with previous findings in other cell types where an increase in elasticity is correlated with an increase in F-actin fluorescent signal [17, 35]. However, it is possible that in microglia, cytoskeletal rearrangements (similar to astrocytes [21]) and not an increase in F-actin per se are responsible for the changes in elasticity. This is also supported by data indicating that disruption of F-actin fiber organization, and not necessarily a decrease in F-actin, decreases cell elasticity [42]. Further research using high-end confocal imaging techniques is likely needed to elucidate the exact role of the cytoskeleton in microglia elasticity and response to inflammation. In addition, whereas the strongest link to cell elasticity has been shown to be F-actin [17, 21], other cytoskeleton proteins could be of importance to alter the biomechanical properties, depending on the cell type [43].

Although not often studied, viscosity in relation to elasticity is of equal importance to determine cellular biomechanical properties. The decrease in viscosity in both WM- and GM-derived microglia when treated with myelin indicates the easier flow of fluids inside the cells, possibly mediated by an increase in cytoplasm fluid volume also affecting cell elasticity measures. Indeed, an increase in cytoplasm volume is related to a decrease in cell elasticity and vice versa [44]. Thus, in microglia, cytoplasm volume could also be an important indicator of microglial biomechanical properties in concert with cytoskeletal proteins. We subsequently determined to what extent inflammatory conditions will have a differential impact on biomechanical properties of WM- and GM-derived microglia and mRNA level changes. Treatment of the cells with LPS, as a typical example of a pro-inflammatory stimulus, resulted in a decrease in cell elasticity and viscosity in WM-derived microglia which resembled observations in LPS-treated macrophages [19, 37, 45]. In those macrophages, the decrease in cell elasticity induced by LPS was accompanied by increased release of pro-inflammatory cytokines [19, 45]. In line with this observation, in WM-derived microglia, the decrease in cell elasticity and viscosity coincided with an increase in *Il-1β* and *Il-6* mRNA, but not *Tnfα mRNA* levels. In contrast, in GM-derived microglia, LPS treatment increased cell elasticity and viscosity making them more resistant to deformation and fluid-like, which coincided with an increase in *Il1-β* and *Il-6* mRNA but also in *Tnfα* mRNA levels. This increase in *Tnfα* mRNA solely in GM-derived microglia could be indicative of a neuroprotective signature of these microglia. Preventing neuronal loss upon brain damage (i.e., inflammation or demyelination) is crucial to maintain proper brain functioning, and microglial-derived TNFα has been implicated in preventing neuronal cell death [46]. In addition, the binding of Tnfα to the TNF receptor 2, which is expressed by neurons, is related to remyelination [47, 48]. Thus, our data possibly suggest that in response to inflammation, i.e., LPS, GM-derived microglia increase their cell elasticity and viscosity to selectively facilitate the production and possibly release of TNFα to prevent neuronal cell death. The absence of an increase in *Tnfα* mRNA production in WM-derived microglia could be related to their relative reduced elasticity and viscosity when treated with LPS, as Tnfα production but not IL-1β is hindered when cell elasticity and viscosity is reduced [49, 50]. The effect of cell elasticity or viscosity on IL-6 production has not been studied yet, but *Il-6* mRNA is reduced in macrophages cultured on stiffer substrates compared to softer substrates [51]. Thus, our data suggest that the production of cytokines can be differentially regulated by cell biomechanical properties.

The loss of myelin is a pathological hallmark of MS. This myelin will be phagocytized by macrophages and local microglia. As infiltrating macrophages are relatively absent in GM lesions [2, 3], we were interested to see how treatment with myelin affects the biomechanical properties of WM- and GM-derived microglia. Upon treatment with myelin, WM- and GM-derived microglia decreased their elasticity and viscosity making them both less resistant to deformation and more fluid-like. This is of interest as it has been reported that a decrease in cell elasticity may facilitate phagocytosis [19]. Indeed, we observed clear myelin phagocytosis in both WM- and GM-derived microglia. In the presence of inflammatory LPS, GM-derived microglia phagocytized more myelin than WM microglia. Although translation to MS is difficult, this may suggest increased removal of myelin debris from GM lesions by microglia if inflammation is present. Although myelin-treated WM and GM-derived microglia showed similar biomechanical properties, only GM-derived microglia showed significantly upregulated expression of the MHC class II antigen expression molecule *Hla-dr* and elevated mRNA levels of corresponding *Cd74* and MHC class I-related *B2m*. In situations resembling demyelination in MS, where there is an abundance of myelin together with inflammation (myelin+LPS condition), WM-derived microglia showed no increase of *Hla-dr* and a decreased cell elasticity and viscosity, possibly hindering the production of Tnfα mRNA as is also observed before [49]. This lack of *Hla-dr* and *Tnfα* expression by microglia might interfere with the ability of the area to remyelinate, as both have been shown to increase oligodendrocyte proliferation [47, 52]. Instead, in GM-derived microglia, we observed a phenotype more closely resembling that of microglia facilitating remyelination, possibly again to support neurons, with increased *Hla-dr* and *Tnfa* mRNA levels and no net change in cell elasticity or viscosity in the myelin + LPS condition. Our results are in contrast with the pathological characteristics of WM- and GM-demyelinated areas: In tissues, it is WM-demyelinated areas that feature more HLA-DR expression, not GM-demyelinated areas [3]. However, it is yet unclear if it is microglia or infiltrated myeloid cells that express HLA-DR in these lesions [5, 53]. Our results possibly suggest that it could be primarily infiltrated monocytes, not microglia, that express HLA-DR in (active) WM-demyelinated areas as we observed no large increase in HLA-DR expression in WM-derived microglia. Thus, the differences between WM- and GM-derived microglia, both at the biomechanical and the biochemical (i.e., mRNA levels), are primarily related to a different immune response to LPS or myelin.

## Conclusions

Our study put forward a biomechanical dimension of microglia heterogeneity as captured by dynamic indentation. Taken together, these data suggest a possible role of biomechanical in concert with biochemical microglia properties which could together determine the characteristics of microglia in MS WM and GM. We are the first to show that at baseline and after treatment with pro-inflammatory LPS, microglia derived from the WM and GM show differential biomechanical characteristics and cytokine expression profiles and can thus likely be considered different subpopulations. However, this heterogeneity in biomechanical characteristics is not found upon exposure to myelin though we do observe differences in mRNA levels of, i.e., *Hla-dr.* Thus, in demyelinating conditions, when myelin debris is phagocytized, as in MS lesions, it is likely that the observed differences in WM- versus GM-derived microglia biomechanics are mainly due to a difference in response to inflammation, rather than to the event of demyelination itself.

## Supplementary Information

**Additional file 1: Figure S1**. Cell depth in μm of WM (blue, left of dotted line) and GM (red, right of dotted line) enriched microglia after treatment with LPS, myelin or myelin+LPS. Black dots represent data from individual microglia, bars represent the mean and +/- SEM. **Figure S2**. Representative lightmicroscopical image of primary mixed glial cells cultured in ibidi μ-dish with grid used for indentation. Microglia are identified by IBA-1 immunoreactivity (pink), astrocytes are identified by GFAP immunoreactivity (brown). Scalebar = 250 μm. **Figure S3**. Graph depicting mRNA levels of Aif-1 and Gfap in WM (blue) and GM (red) derived mixed glial cell cultures. Black dots represent data from 3 individual cultures, bars represent the mean and +/- SEM. We observed no statistical differences in the mRNA expression of Aif-1 or Gfap between conditions (ns).

## Data Availability

The datasets used and/or analyzed during the current study are available from the corresponding author on reasonable request.

## Abbreviations

WM: White matter
GM: Gray matter
LPS: Lipopolysaccharide
MS: Multiple sclerosis
EAE: Experimental autoimmune encephalitis
MHC-II: Major histocompatibility complex class II
