# Supplementary material for: Viscoelastic properties of white and gray matter-derived microglia differentiate upon treatment with lipopolysaccharide but not upon treatment with myelin
Source: J Neuroinflammation. 2021 Mar 29;18:83. doi: 10.1186/s12974-021-02134-x (PMC8008683; doi:10.1186/s12974-021-02134-x)
Supplement: Supplementary file 1 — Additional file 1: Figure S1. Cell depth in μm of WM (blue, left of dotted line) and GM (red, right of dotted line) enriched microglia after treatment with LPS, myelin or myelin+LPS. Black dots represent data from individual microglia, bars represent the mean and +/- SEM. Figure S2. Representative lightmicroscopical image of primary mixed glial cells cultured in ibidi μ-dish with grid used for indentation. Microglia are identified by IBA-1 immunoreactivity (pink), astrocytes are identified by GFAP immunoreactivity (brown). Scalebar = 250 μm. Figure S3. Graph depicting mRNA levels of Aif-1 and Gfap in WM (blue) and GM (red) derived mixed glial cell cultures. Black dots represent data from 3 individual cultures, bars represent the mean and +/- SEM. We observed no statistical differences in the mRNA expression of Aif-1 or Gfap between conditions (ns). [file 12974_2021_2134_MOESM1_ESM.pdf]

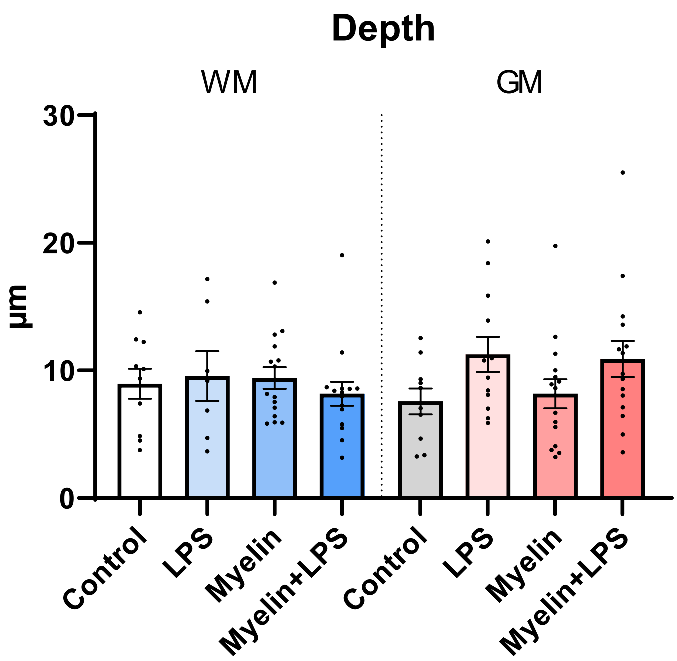

Supplementary Figure 1: Cell depth in µm of WM (blue, left of dotted line) and GM (red, right of dotted line) enriched microglia after treatment with LPS, myelin or myelin+LPS. Black dots represent data from individual microglia, bars represent the mean and +/- SEM.

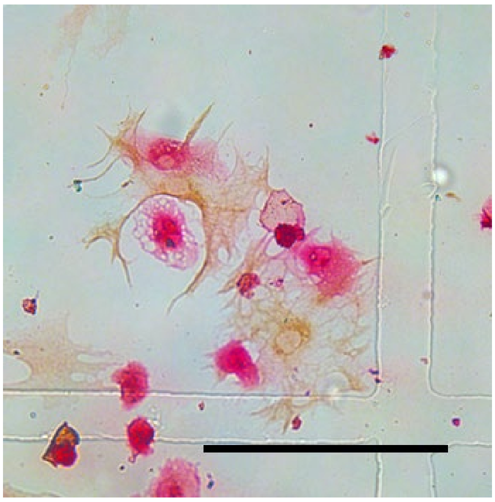

Supplementary Figure 2: Representative lightmicroscopical image of primary mixed glial cells cultured in ibidi µ-dish with grid used for indentation. Microglia are identified by IBA-1 immunoreactivity (pink), astrocytes are identified by GFAP immunoreactivity (brown). Scalebar = 250 µm.

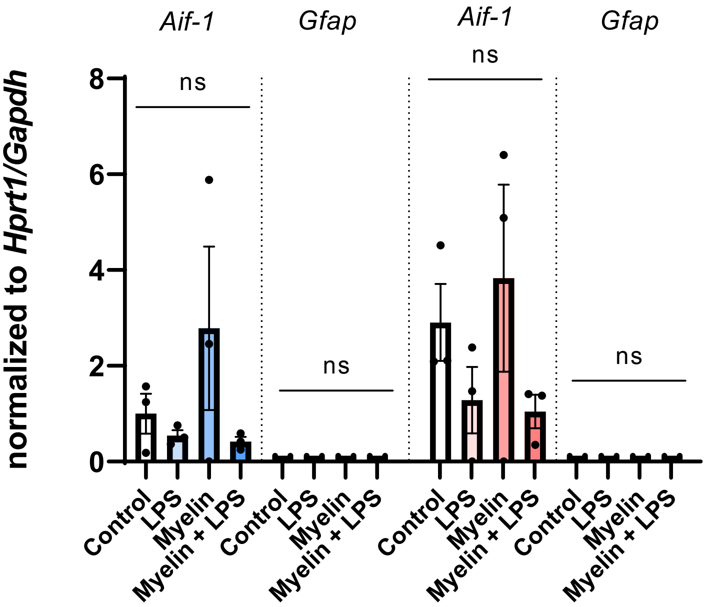

Supplementary Figure 3: Graph depicting mRNA levels of *Aif-1* and *Gfap* in WM (blue) and GM (red) derived mixed glial cell cultures. Black dots represent data from 3 individual cultures, bars represent the mean and +/- SEM. We observed no statistical differences in the mRNA expression of *Aif-1* or *Gfap* between conditions (ns).
